# *PARP1* gene expression is downregulated by knockdown of *PARG* gene

**DOI:** 10.3892/or.2013.2321

**Published:** 2013-03-04

**Authors:** FUMIAKI UCHIUMI, TAKESHI WATANABE, RYO OHTA, HIDEAKI ABE, SEI-ICHI TANUMA

**Affiliations:** 1 Department of Gene Regulation, Faculty of Pharmaceutical Sciences, Tokyo University of Science, Noda-shi, Chiba-ken 278-8510, Japan; 2 Department of Biochemistry, Faculty of Pharmaceutical Sciences, Tokyo University of Science, Noda-shi, Chiba-ken 278-8510, Japan; 3 Genome and Drug Research Center, Tokyo University of Science, Noda-shi, Chiba-ken 278-8510, Japan; 4 Research Center for RNA Science, RIST, Tokyo University of Science, Noda-shi, Chiba-ken 278-8510, Japan

**Keywords:** poly(ADP-ribose), poly(ADP-ribose) glycohydrolase, poly(ADP-ribose) polymerase, short interfering RNA, Sp1

## Abstract

Poly(ADP-ribosyl)ation is a modification of nuclear proteins that regulates DNA replication, repair and transcription. In order to investigate the biological effects of degradation of poly(ADP-ribose), knockdown of the poly(ADP-ribose) glycohydrolase (*PARG*) gene was performed by introducing a short interfering RNA (siRNA)-pool into HeLa S3 cells. Notably, poly(ADP-ribosyl)ated proteins did not accumulate in the cells. Western blotting, quantitative RT-PCR analysis and a transient transfection assay revealed that poly(ADP-ribose) polymerase 1 (*PARP1)* gene/protein expression and its promoter activity were reduced in the *PARG* knockdown cells. These results suggest that the amount of poly(ADP-ribose) in a cell is regulated under the control of *PARP1/PARG* gene expression balance. Furthermore, in this study, we showed that *PARG*-siRNA enhanced cell death induced by staurosporine (STS). Thus, we propose a *PARG*-siRNA utilizing gene-therapy for cancer treatment.

## Introduction

Poly(ADP-ribosyl)ation is a NAD^+^-dependent post-transcriptional modification of chromosomal proteins mediated by poly(ADP-ribose) polymerases (PARPs) and poly(ADP-ribose) glycohydrolase (PARG) (1). Reversible poly(ADP-ribosyl)ation of chromosomal proteins has been suggested to play important roles in various biological processes, including DNA replication (2,3), repair (4–6), spindle assembly (7), transcription (8), telomere and chromosomal maintenance (9) and epigenetic gene regulation (10). Previous studies have suggested that poly(ADP-ribose) metabolism is associated with differentiation and proliferation (11,12), cell death (13) and apoptosis (14,15). Thus, poly(ADP-ribosyl)ation is significantly involved in various biological activities, suggesting that it requires precise controlling systems to adjust the amount and length of poly(ADP-ribose) in eukaryotic cells.

Previous studies indicated that several transcription factors, including Sp1 (16), YY1 (17), and NF-κB (18), are poly(ADP-ribosyl)ated and reduce the transcription activity (16). If there are binding sites for these transcription factors in the promoter region, transcription might be affected by the poly(ADP-ribosyl)ation. To date, the promoter regions of the human *PARP1*(19) and *PARG*(20,21) genes have been isolated and characterized. In this study, we investigated the effect of short interfering RNAs (siRNAs) for the human *PARG* cDNA on poly(ADP-ribosyl)ation and *PARP1* gene expression in HeLa S3 cells. The results showed that expression and promoter activity of the *PARP1* gene were reduced by the knockdown of the *PARG* gene and that the amounts of poly(ADP-ribose) in the cells did not increase compared to the control cells. Moreover, *PARG* knockdown cells showed stronger cell death sensitivity to staurosporine (STS) than the control cells, suggesting that retarded turnover of poly(ADP-ribose)-NAD^+^ metabolism might induce intracellular apoptosis signals.

It is well known that PARP1 activity is downregulated by its augmented auto-poly(ADP-ribosyl)ation (22,23), and artificially accumulated poly(ADP-ribose) induces apoptosis (13). Collectively, our results indicate that reduced poly(ADP-ribose) degradation subsequently suppresses transcription of the *PARP1* gene to escape excessive poly(ADP-ribose) accumulation, thereby achieving a balance in poly(ADP-ribose) levels for cell survival. Therefore, poly(ADP-ribose) may act as a dual regulator for PARP1 activity not only at the post-translational level but also at the transcriptional level. Hence, we propose a molecular mechanism that prevents cells from accumulating excess amounts of poly(ADP-ribose) by regulating transcription of the *PARP1* gene.

## Materials and methods

### Cell culture

Human cervical carcinoma (HeLa S3) cells (24) were grown in Dulbecco’s modified Eagle’s medium (DMEM; Nacarai, Tokyo, Japan), supplemented with 10% fetal bovine serum (FBS) (Sanko Pure Chemicals, Tokyo, Japan) and penicillin-streptomycin at 37°C in a humidified atmosphere with 5% CO_2_.

### Transfection of siRNA

The ON-TARGETplus SMARTpool siRNAs used for knockdown of the human *PARG* gene were purchased from Thermo Fisher Scientific Inc. (Lafayette, CO, USA). They were introduced into HeLa S3 cells with DharmaFECT Transfection reagent following the manufacturer’s protocol (Thermo Fisher Scientific). In brief, 2 μM siRNA (50 μl) were added to serum-free DMEM (50 μl) in one tube, and DharmaFECT1 (1.5 μl) was added to 98.5 μl of serum-free medium in the other tube. They were gently mixed and incubated for 5 min at room temperature, and were then combined, mixed and further incubated for 20 min at room temperature. Subsequently, complete medium (800 μl) was added and cells were cultivated with the medium in a 35-mm culture dish.

### Cell viability MTS assay

An MTS assay was performed according to the manufacturer’s instructions. In brief, mock- or siRNA-transfected cells were cultured in microtiter plate wells. MTS solution (20 μl) (Promega, Madison, WI, USA) was added to each well (containing 100 μl of cell culture) and incubated for 3 h in a 37°C, 5% CO_2_-humidified incubator. Then, the absorbance at 492 nm was measured by a microtiter plate reader (Thermo Electron Corp., Vantaa, Finland) and normalized by the absorbance at 630 nm.

### Reverse transcriptase and quantitative real-time polymerase chain reaction (RT-qPCR)

RT-qPCR was carried out as previously described (24). First-strand cDNAs were synthesized with ReverTra Ace (Toyobo Corp., Tokyo, Japan), random primers (Takara, Kyoto, Japan) and total RNAs were extracted from HeLa S3 cells. A primer pair to amplify the human *GAPDH* cDNAs was previously reported (24), and those for amplifying the *PARP1* and *PARG* cDNAs were: hPARP1S514, 5′-GCAGAGTATGCCAAGTCCAACAG-3′ and hPARP1AS813, 5′-ATCCACCTCATCGCCTTTTC-3′; and hPARG-S, 5′-ATGTGTAAGTGGCAAAATGAAGGG-3′ and hPARG-A952, 5′-CTTCTCTGGCCTGTTCATCTTC-3′, respectively. Real-time PCR analysis was carried out using the Mx3000P Real-Time QPCR system (Stratagene, La Jolla, CA, USA) as previously described (24). For PCR amplification, cDNAs were amplified using SYBR-Green real-time PCR Master Mix (Toyobo) and 0.3 μM of each primer pair. Amplification of the *PARP1* cDNA was carried out, starting with an initial step for 1 min at 94°C, followed by 42 cycles (94°C 30 sec, 55°C 30 sec and 72°C 1 min). The conditions for amplification of the *PARG* and *GAPDH* cDNAs were 1 min at 94°C, followed by 42 cycles (94°C 15 sec, 55°C 10 sec, and 72°C 15 sec).

### Western blot analysis

Western blotting was carried out as previously described (24,25) with antibodies against PARP1 (Santa Cruz Biotechnology, Santa Cruz, CA, USA) and PAR (Calbiochem, Darmstadt, Germany) followed by the addition of horseradish peroxidase (HRP)-conjugated secondary antibody (Calbiochem). Signal intensities were quantified with a LAS4000 system and Multi Gauge Software (Fuji Film, Tokyo, Japan).

### Construction of luciferase (Luc) reporter plasmids

Luc reporter plasmids carrying 75-bp of the human PARG promoter regions were designated pKBST-Δ6 (21). The 5′-flanking regions of the human *PARP1* gene were obtained by PCR with PrimeStar Taq polymerase (Takara) and the template genomic DNA from HeLa S3 cells as previously described (26). The sense and antisense primers used for PCR were: hPARP1-2660, 5′-TCGGTACCGGGTCCTCCAAAGAGCTAC-3′; and AhPARP1-2895, 5′-ATCTCGAGCCGCCACCGAACACGC CGC-3′, respectively. The amplified DNA fragments were digested with *Kpn*I and *Xho*I and ligated into the MCS of the pGL4-basic (pGL4.10[luc2]), vector (Promega) to make pGL4-PARP1. Deletion derivatives, pGL4-PARP1Δ1 and pGL4-PARP1Δ2, were generated by PCR with pGL4-PARP1 as the template and primer sets: hPARP1-2851, 5′-TCGGTA CCGCCAGGCATCAGCAATCTA-3′ and AhPARP1-2895; and hPARP1-2660 and AhPARP1-2851, 5′-ATCTCGAGTA GATTGCTGATGCCTGGC-3′, respectively. The nucleotide sequences of the PCR products were determined by a DNA Sequencing system (Applied Biosystems, Foster City, CA, USA) with Rv (5′-TAGCAAAATAGGCTGTCCCC-3′) and GL (5′-CTTTATGTTTTTGGCGTCTTCC-3′) primers.

### Transient transfection and Luc assays

Plasmid DNAs were transfected into HeLa S3 cells by the DEAE-dextran method (24–26). After a further 24 h of incubation, cells were collected and lysed with 100 μl of 1X cell culture lysis reagent, mixed and centrifuged at 12,000 × g for 5 sec. The supernatant was stored at −80°C. The Dual Luciferase assay was performed with the Dual Luciferase assay system (Promega), as previously described (21).

## Results

### Decrease in the amounts of PARG gene transcripts after introducing its siRNAs

**Figure 1:**
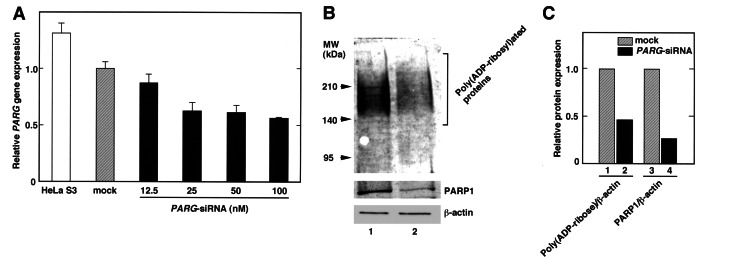
The effect of knockdown of the *PARG* gene by introducing siRNA into HeLa S3 cells. (A) Quantitative real-time PCR analysis was performed with total RNAs isolated from HeLa S3 cells after 48 h of transfection with 0 (mock), 12.5, 25, 50 or 100 nM of *PARG*-siRNA. Results show the relative expression of the *PARG* gene compared to the *GAPDH* gene. The data are shown as the means ± SEM of three independent experiments. (B) HeLa S3 cells were transfected with 0 or 100 nM of *PARG*-siRNA (lanes 1 and 2, respectively). After 48 h of incubation, proteins were extracted from cells and separated by SDS-PAGE, then western blotting was performed with antibodies against poly(ADP-ribose), PARP1 and β-actin (upper, middle and lower panels, respectively). (C) Each band was quantified and results show relative PAR/β-actin and PARP1/β-actin expression ratios compared with those of the mock-transfected cells.

In order to suppress *PARG* gene expression, ON-TARGETplus SMARTpool siRNAs (*PARG*-siRNA) were transfected into HeLa S3 cells with DharmaFECT 1 reagent. The relative amounts of PARG transcripts were reduced by *PARG*-siRNA treatment (Fig. 1A). In 100 nM of *PARG*-siRNA-treated cells, the PARG gene expression level decreased to approximately half of that in the mock-transfected cells. Since this treatment did not affect viability of cells compared with the mock-transfected cells (data not shown), further experiments were performed with 100 nM of *PARG*-siRNA.

### Treatment of PARG-siRNA reduces the amounts of poly(ADP-ribose) and PARP1

As the *PARG* gene encodes the main poly(ADP-ribose) degrading enzyme, the level of poly(ADP-ribosyl)ated proteins was assumed to increase following the introduction of *PARG*-siRNA into HeLa S3 cells. Western blotting revealed that the amounts of poly(ADP-ribose) decreased in the *PARG*-siRNA-transfected HeLa S3 cells (Fig. 1B). In addition, the decrease of poly(ADP-ribose) in the whole-cell extracts was accompanied by a decrease in PARP1 protein levels (Fig. 1C), suggesting that the *PARG*-siRNA diminished poly(ADP-ribose) and the PARP1 protein.

### PARP1 gene expression and its promoter activity are downregulated by transfection with PARG-siRNA

**Figure 2:**
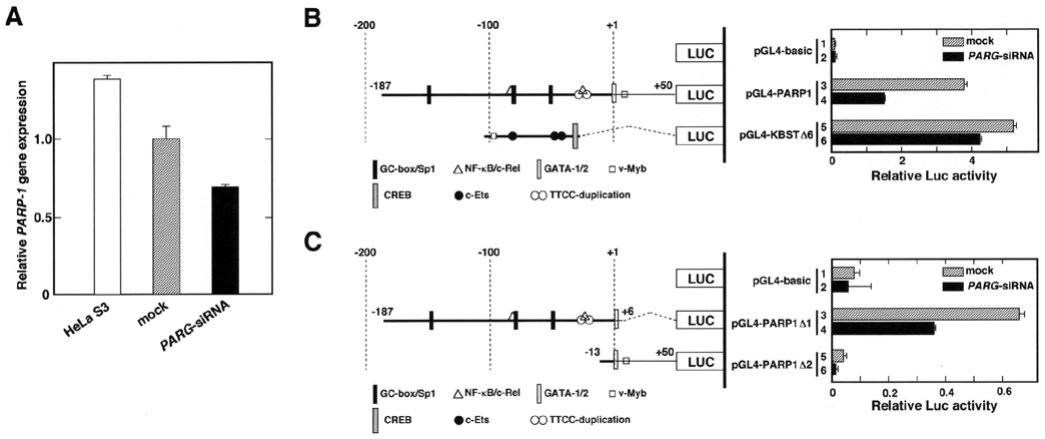
*PARG* knockdown leads to downregulation of *PARP1* gene expression and its promoter activity. (A) HeLa S3 cells were transfected with 100 nM of the *PARG*-siRNA. After 48 h of incubation, total RNAs were isolated and subjected to real-time qPCR analysis with *PARP1*- or *GAPDH*-specific primer pairs. Histograms show relative expression of the *PARP1* gene compared to the *GAPDH* gene. The data are shown as the means ± SEM of three independent experiments. (B) HeLa S3 cells were transfected with 100 nM of the *PARG*-siRNA. After 48 h of incubation, cells were transfected with Luc-reporter plasmids, pGL4-basic (bars 1 and 2), pGL4-PARP1 (bars 3 and 4), and pGL4-KBSTΔ6 (bars 5 and 6). After a further 24 h of incubation, cells were harvested and a dual Luc assay was carried out. (C) Similar experiments were carried out by transfecting pGL4-basic (bars 1 and 2), pGL4-PARP1Δ1 (bars 3 and 4), and pGL4-PARP1Δ2 (bars 5 and 6). (B and C) Histograms show the relative Luc activities compared to cells transfected with the pGL3-promoter vector. The data are shown as the means + SEM of three independent experiments.

To examine whether the decrease in PARP1 protein level is caused by changes in gene expression and promoter activity, RT-qPCR analyses and transient transfection experiments were performed. As shown in Fig. 2A, the relative *PARP1* gene expression of *PARG*-siRNA-transfected cells was 60% that of the control (mock) cells. In addition, the transient transfection and Luc reporter assay indicated that the *PARP1* promoter activity decreased in the *PARG*-siRNA-transfected cells compared to the control cells (Fig. 2B). We also tested whether the 75-bp core promoter of the *PARG* gene (21) responds to *PARG*-siRNA treatment (Fig. 2B, bars 5 and 6), but only a 10% decrease was observed. To limit the *PARG*-siRNA responsive element(s), we constructed two deletion plasmids that contained −187 to +6 and −13 to +50 bp of the human PARP1 promoter (Fig. 2C). The results showed that the 193-bp fragment, which contains the GC-box/Sp1, NF-κB/c-Rel, GATA-1 and GATA-2 binding sequences, responded to the *PARG*-siRNA. The promoter activity obtained from pGL4-PARP1Δ2-transfected cells was lower than that of the pGL4-basic vector-transfected cells, indicating that the 63-bp sequence from −13 to +50 is not essential for transcription of the *PARP1* gene. These results suggest that the transfection of *PARG*-siRNA leads to suppression of *PARP1* gene expression through the 193-bp sequence of the *PARP1* promoter region.

### Viability of PARG siRNA-introduced HeLa S3 cells following N-methyl-N′ nitro-N-nitrosoguanidine (MNNG) and STS treatments

**Figure 3:**
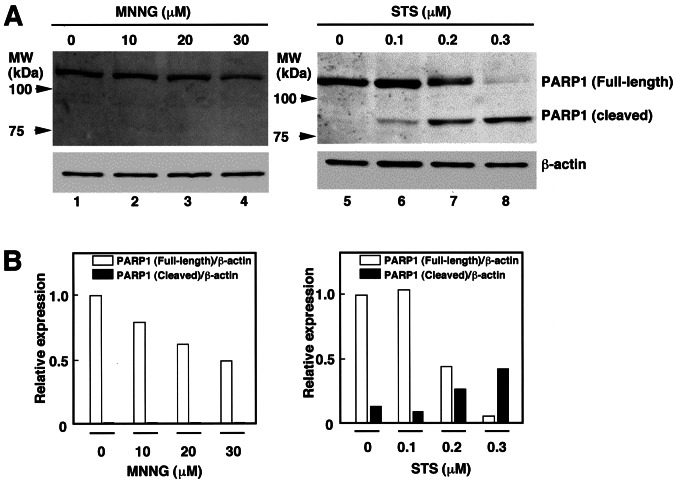
Effect of N-methyl-N′ nitro-N-nitrosoguanidine (MNNG) and staurosporine (STS) on PARP1 cleavage in HeLa S3 cells. (A) HeLa S3 cells (1×10^6^) were treated with 10, 20 and 30 μM of MNNG (lanes 2, 3 and 4, respectively), or 0.1, 0.2 and 0.3 μM of STS (lanes 6, 7 and 8). After 24 h of incubation, cells were harvested, and whole protein extract was prepared with RIPA buffer, which was then subjected to SDS-PAGE. Western blotting was performed with antibodies against PARP1 (upper panels) and β-actin (lower panels). Lanes 1 and 5 represent untreated control cells. (B) Signal intensities of full-length (open columns) and cleaved-form (closed columns) PARP1 were quantified and normalized to β-actin. Histograms show the relative protein level compared to that of the full-length PARP1/β-actin ratio estimated from control cells.

It has been demonstrated that poly(ADP-ribose) metabolism is associated with cell death or apoptosis (13–15). To examine the effects of MNNG and STS on PARP1 in HeLa S3 cells, western blot analysis was performed (Fig. 3). The treatment with STS (0.1–0.3 μM) induced cleavage of PARP1, suggesting that caspases were activated and cells underwent apoptosis. Although the relative amount of PARP1 was diminished to 50%, cleaved forms of PARP1 were not detected by the treatment with 30 μM of MNNG (Fig. 3).

**Figure 4:**
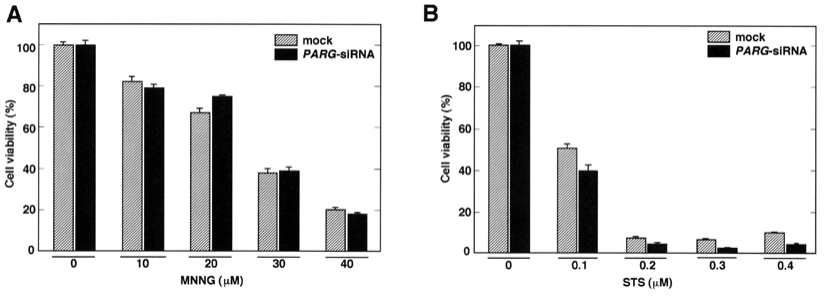
Effect of *PARG* knockdown on the sensitivity to cell death-inducing reagents. After 48 h of transfection with *PARG*-siRNA (100 nM), (A) MNNG or (B) STS was added to the culture medium. After a further 24 h of incubation, an MTS assay was carried out. The data are shown as the means ± SEM of three independent experiments. MNNG, N-methyl-N’ nitro-N-nitrosoguanidine; STS, staurosporine.

Subsequently, the effect of MNNG and STS on cell viability was analyzed following the transfection of *PARG*-siRNA. Although 40 μM of MNNG treatment caused severe damage to the cells, transfection of *PARG*-siRNA did not further increase cell death (Fig. 4A). On the other hand, *PARG*-siRNA strongly impaired cell viability when cells were treated with 0.3 and 0.4 μM of STS (Fig. 4B). These results suggest that *PARG*-siRNA treatment enhances cell death in conditions that cause PARP1 cleavage.

## Discussion

Several experiments concerning expression of the *PARG* gene that have utilized *PARG*-siRNA or *PARG*-shRNA overexpressing systems have been reported (27). Constitutive suppression of *PARG* gene expression by introducing shRNA into HeLa S3 cells caused accumulation of poly(ADP-ribose) and led to enhancement of mitotic catastrophe by X-ray irradiation (28). It was reported that poly(ADP-ribose) accumulation occurred after a *PARG* shRNA expression vector was stably introduced into A549 cells, a lung adenocarcinoma cell line (29). Of note, these PARG-suppressed A549 cells exhibited reduced PARP activity. The decrease in poly(ADP-ribose) was reported in mouse 3T3 embryonic fibroblasts, PARG-Δ2,3 cells, which express PARG60 but not full-length PARG110 (30). In the present study, a *PARG*-siRNA pool that contains sequences located upstream of exon 5 was transfected into HeLa S3 cells. Therefore, *PARG*-siRNA used here may selectively reduce full-length PARG expression but may not affect PARG60. In addition, the suppressing effect by the introduction of 25–100 nM of the *PARG*-siRNA was approximately 50% of the control (Fig. 1A). This low efficiency might be caused by the reduced poly(ADP-ribose) level in the cells (Fig. 1B).

It has been suggested that poly(ADP-ribosyl)ation of Sp1 impairs activation of the *PARP1* promoter (16). It has also been reported that PARP1 binds to its own promoter to suppress transcription (31). Since PARP1 physically interacts with Sp1 (16), both may cooperatively regulate transcription of the *PARP1* gene. In our study, the introduction of *PARG*-siRNA into HeLa S3 cells caused suppression of the *PARP1* promoter activity and led to subsequent reduction of its gene/protein expression. These data suggest a possible mechanism that prevents cells from accumulating excess poly(ADP-ribose) by suppressing *PARP1* promoter activity. It is noteworthy that inverted repeats or putative cruciform-like structures are found both in the *PARP1*(31) and *PARG*(21) promoters. Since PARP1 has been suggested to recognize and bind to the cruciform-like structures of DNA (31), the *PARG* promoter is possibly one of the targets for PARP1. Moreover, the *PARP1* promoter harbors overlapped TTCC motifs as 5′-GCGGG**TT CC**GTGGGCG**TTCC**CGCGG-3′ (Fig. 2B and C). The duplicated GGAA (TTCC) motifs, which are also found in the human *PARG* promoter region (21), have been suggested to be one of the determinants of TSS in TATA-less promoters with responses to various stimuli, including cytokine and differentiation inducing signals (32–34). In addition, previous reports suggested that PARP1 and PARG interact with each other (14) and coordinately regulate global patterns of gene expression, and that the binding of PARP1 to the promoters in the genome is dependent on the presence of PARG (27). Collectively, these observations indicate that the location of PARP1 with PARG on these promoters may play an important role in the regulation of poly(ADP-ribose) metabolism at the transcriptional level. This might be relevant to the cooperative function of PARP1 and PARG to repair single-strand break of DNA (35).

In this study, we showed that the introduction of *PARG-*siRNA enhances sensitivity to STS but not to MNNG (Fig. 4). Since PARP1 cleavage was detected in STS-treated cells (Fig. 3), the introduction of *PARG*-siRNA may cause elevation of cell damage by reducing PARP1 expression and lead to complete loss of native PARP1, and induce cell death by apoptosis. On the other hand, cell death induced by MNNG was not further affected by the *PARG*-siRNA, suggesting that the signals induced by the reduction of PARP1 have been adequately saturated by the MNNG treatment. However, this possibility remains to be elucidated in future analyses.

In conclusion, the data presented in this study provide a new theory that *PARP1* gene expression is regulated by the amount of *PARG* transcripts, suggesting a mechanism to avoid accumulation of excess amounts of poly(ADP-ribose) in a cell by regulating their transcription. Since poly(ADP-ribose) metabolism also controls NAD^+^ levels, local changes in the concentrations of NAD^+^ might affect transcription of the *PARP1* and *PARG* genes. Markedly, in accordance with the development of PARG inhibitors (36), the use of the *PARG*-siRNA may contribute to a treatment of cancer with a reduced dose of anti-cancer drugs or ionizing radiation.
